# Ten‐year trends in hospitalizations due to Alzheimer's disease in South west of China

**DOI:** 10.1002/alz70860_101758

**Published:** 2025-12-23

**Authors:** Xueping Chen

**Affiliations:** ^1^ west china hospital, Chengdu, Sicuan, China; West China Hospital, Sichuan University, Chengdu, Sichuan, China

## Abstract

**Background:**

Alzheimer's disease (AD) is the most common neurodegenerative disorder. The present study evaluated the AD hospitalization profile in China between 2011 and 2021 in a tertiary medical center.

**Method:**

The study used analytical, observational, and retrospective methods, utilizing data extracted from the Hospital Information Systems of the West China Hospital.

**Result:**

From 2011 to 2021, 2187 patients were hospitalized due to AD or its comorbidities. The presence of comorbid conditions caused 53.41% hospitalizations, and the comorbidities for hospital admission included infection (10.70%), internal medical disease (7.18%), psychiatric disorders (6.45%), and fracture (4.80%). Non‐comorbid hospitalization and neurological admission were protective factors for poor outcomes in hospitalized AD patients.

**Conclusion:**

The current study helps enhance our understanding of AD hospitalization patterns in China, and a comprehensive intervention might be a practical approach to reduce hospitalization for AD patients and create public health policies focused on reducing the costs linked to AD hospitalization.

